# Editorial: Telemedicine during and beyond COVID-19, volume II

**DOI:** 10.3389/fpubh.2022.1057879

**Published:** 2022-11-04

**Authors:** Sonu M. M. Bhaskar, Alma Nurtazina, Shikha Mittoo, Maciej Banach, Robert Weissert

**Affiliations:** ^1^Pandemic Health System REsilience PROGRAM (REPROGRAM) Global, Sydney, NSW, Australia; ^2^Global Health Neurology Lab, Sydney, NSW, Australia; ^3^Neurovascular Imaging Laboratory, Ingham Institute for Applied Medical Research, Clinical Sciences Stream, Sydney, NSW, Australia; ^4^NSW Brain Clot Bank, NSW Health Pathology, Sydney, NSW, Australia; ^5^Liverpool Hospital & South West Sydney Local Health District (SWSLHD), Department of Neurology & Neurophysiology, Sydney, NSW, Australia; ^6^Department of Epidemiology and Biostatistics, Semey Medical University, Semey, Kazakhstan; ^7^Department of Rheumatology, University Health Network and The University of Toronto, Toronto, ON, Canada; ^8^Department of Cardiology and Congenital Diseases of Adults, Polish Mother's Memorial Hospital Research Institute, Lodz, Poland; ^9^Cardiovascular Research Centre, University of Zielona Góra, Zielona Gora, Poland; ^10^Department of Hypertension, Medical University of Lodz, Lodz, Poland; ^11^Department of Neurology, University of Regensburg, Regensburg, Germany

**Keywords:** telemedicine, COVID-19, digital health, policy, public health, health systems

COVID-19 is placing an unprecedented strain on health systems in general, and healthcare delivery in particular (1–5). Health systems and services are being reconfigured to maintain patient continuity. Telemedicine has provided an enabling tool to maintain patient continuity and management of patients during COVID-19 (2). It has allowed limiting exposure to virus among patients, healthcare workers and systems which is critical in controlling the spread and transmission. COVID-19 has arguably accelerated the adoption of telemedicine. However, there are challenges, including telemedicine-specific infrastructure and technology in remote settings, language barriers, and regulatory and other issues, which call for comprehensive evaluation (5). Despite technological advances in telemedicine and urgency in its uptake during COVID-19, language and socioeconomic barriers relevant to vulnerable communities pose an ongoing challenge (6, 7).

In the second edition of the Research Topic on “*Telemedicine during and beyond COVID-19*,” we highlight the consequences and changes in telemedicine caused by and following the pandemic. This includes not only the status of telemedicine in developed countries, but also discussions around its adoption across the geographical divide, specific gaps that apply to medical specialities such as pediatrics, and future areas of research and development, among others. The current pandemic affects diagnostic workup, medical treatment, and surgical procedures, as well as chronic management of diseases (4). Telemedicine is critical for pandemic preparedness and action during and beyond COVID-19. In the current volume, we intended to explore the impact telemedicine has made with a focus on the latter stages of the pandemic.

Mahmoud et al. performed a scoping review to study the extent and acceptance of telemedicine in low- and middle-income countries (LMICs) during COVID-19. Authors identified infrastructure and regulatory barriers as the most significant barriers to telemedicine use in LMICs. The authors also concluded that telemedicine improved access to high-quality healthcare and mitigated infection risk during COVID-19.

Lan et al. set out to investigate the research direction of telemedicine during COVID-19 by performing a bibliometric analysis to understand what type of telemedicine technology is employed in which diseases and what medical services are given by telemedicine. The study validated the wide use of telemedicine during COVID-19.

Michel, Mettler et al. share their experiences with the COVID-19 online forward triage tool (OFTT) in pediatric settings in Switzerland. The authors concluded that it is essential that stakeholders, parents, teachers, and healthcare professionals participate in the development, setup, execution, and assessment of telehealth interventions as this can aid in managing expectations and increase the usefulness of OFTT. In another work, the authors explored attitudes, experiences, and challenges faced by OFTT users and their families, concerning public health recommendations (Michel, Rehsmann et al.). It appears that a wide range of interrelated elements affect how people feel about public health advice, which necessitates the use of systems thinking in public health communication. The authors also investigated the health care and health authority perspectives on the utility of OFTT during COVID-19 (Michel, Kilb et al.). The authors aimed to evaluate how this pandemic affected weight reduction *via* a very low-carbohydrate regimen that targeted nutritional ketosis administered *via* telemedicine (NKI). The OFTT's usefulness was found to be hindered by factors such as data privacy, doctor-patient relationships, resistance to change, regulatory and mandate concerns, and a lack of systems thinking.

Another study by Kreider et al. provided an in-depth understanding of the therapeutic reasoning factors, supportive strategies, and practical solutions for telerehabilitation in Veterans. The quick transition of Veterans Health Administration (VHA) healthcare from outpatient rehabilitation services to telerehabilitation during the COVID-19 epidemic expedited gains in practical knowledge for implementing telerehabilitation, which is explained in this study.

Athinarayanan et al. evaluated how COVID-19 affected weight reduction *via* a very low-carbohydrate regimen that targeted nutritional ketosis administered *via* telemedicine (NKI). Independent of pandemic stress and lifestyle restrictions, the authors found that a very low-carbohydrate telemedicine intervention produced comparable and clinically meaningful weight loss.

In a commentary on the neglected digital divide barriers of telemedicine during COVID-19, Cheshmehzangi et al. call for an integrated approach from macro, meso, and micro levels to address these neglected barriers.

The pandemics such as COVID-19 exacerbate health inequities and pressures on health systems and healthcare workers (1, 8). Amidst these challenges, platforms such as telemedicine can be an enabler for health system continuity, preparedness, and action (9, 10). However, systemic barriers or factors linger that compromise its uptake and use. Identifying these barriers, creating evidence-based strategies for standardized telemedicine workups, and ensuring co-design, feedback from users/patients and ongoing monitoring for optimal and equitable use and roll-out will be imperative for our capacity, and sustainability, to respond to future pandemics.

Providing a framework, once health care gaps have been identified and the feasibility of delivery models understood, to health care governments and payors are critical to ensure ongoing real-time adoption of telemedicine platforms tailored to communities at greatest risk for adverse outcomes during and even after any health care crises. Outlining the provisions for telemedicine to such stakeholders also helps to ensure health care access for communities with the greatest health care disparities.

## Author's note

The COVID-19 pandemic is causing an unprecedented public health crisis impacting healthcare systems, healthcare workers, and communities. The COVID-19 Pandemic Health System REsilience PROGRAM (REPROGRAM) consortium is formed to champion the safety of healthcare workers, policy development, and advocacy for global pandemic preparedness and action.

## Author contributions

SB conceptualized and wrote the first draft of the manuscript. SB, AN, MB, SM, and RW discussed the results and recommendations and contributed to the final manuscript. All authors contributed to the article and approved the submitted version.

## Conflict of interest

The authors declare that the research was conducted in the absence of any commercial or financial relationships that could be construed as a potential conflict of interest.

## Publisher's note

All claims expressed in this article are solely those of the authors and do not necessarily represent those of their affiliated organizations, or those of the publisher, the editors and the reviewers. Any product that may be evaluated in this article, or claim that may be made by its manufacturer, is not guaranteed or endorsed by the publisher.
